# Size Effect in Compressive Strength Tests of Cored Specimens of Lightweight Aggregate Concrete

**DOI:** 10.3390/ma13051187

**Published:** 2020-03-06

**Authors:** Lucyna Domagała

**Affiliations:** Faculty of Civil Engineering, Cracow University of Technology, 31-155 Cracow, Poland; ldomagala@pk.edu.pl

**Keywords:** scale effect, specimen size, lightweight concrete, lightweight aggregate, expanded clay, sintered fly ash, compressive strength

## Abstract

The aim of this paper is to discuss the unrecognized problem of the scale effect in compressive strength tests determined for cored specimens of lightweight aggregate concrete (LWAC) against the background of available data on the effect for normal-weight concrete (NWAC). The scale effect was analyzed taking into consideration the influence of slenderness (*λ* = 1.0, 1.5, 2.0) and diameter (d = 80, 100, 125, and 150 mm) of cored specimens, as well as the type of lightweight aggregate (expanded clay and sintered fly ash) and the type of cement matrix (w/c = 0.55 and 0.37). The analysis of the results for four lightweight aggregate concretes revealed no scale effect in compressive strength tests determined on cored specimens. Neither the slenderness, nor the core diameter seemed to affect the strength results. This fact should be explained by the considerably better structural homogeneity of the tested lightweight concretes in comparison to normal-weight ones. Nevertheless, there were clear differences between the results obtained on molded and cored specimens of the same shape and size.

## 1. Introduction

Lightweight aggregate concrete (LWAC) has been one of the most popular and versatile building materials in the world for decades. The most important advantages of its application in comparison to normal-weight concrete (NWAC) of the same strength class are the following:

- Higher thermal insulation and better sound absorption [1,2,3];
- The possibility of building constructions with longer spans and/or higher height and/or smaller cross-sections of structural members [4,5,6];
- The possibility of autogenous shrinkage elimination [7,8,9];
- Better durability: higher fire resistance, possible higher freeze–thaw resistance, possible lower carbonation, and possible lower water permeability [10,11,12,13,14,15,16];
- Less likely to crack, resulting from shrinkage, creep, thermal deformation, or loads [17,18,19,20].

The better durability and lesser likelihood of cracking of LWAC result mostly from the better homogeneity of the LWAC structure. 

Nevertheless, lightweight aggregate concrete is rarely used as a structural material compared to the most popular option—normal-weight concrete. The most important reasons for this situation are some technological problems with LWAC structure execution, i.e., higher risk of workability loss and concrete segregation, as well as a usually higher price per volume unit, and mainly a lack of versatile procedures for designing, execution, testing, and assessment. Meanwhile, the use of structural lightweight concrete, made of manufactured or recycled aggregates, in the near future should become widespread due to the depletion of natural aggregate deposits and the emphasis on sustainable, less energy-consuming constructions.

The size and shape effects of test specimens on the assessment of LWAC’s properties are some of the less qualitatively and quantitatively recognized problems. Generally, according to Griffith’s and Weibull’s theory [3,21], the fracture starts from any critical defect (“the weakest chain”) contained in a material. Therefore, the specimens of larger volumes reveal a higher probability of the presence of such a flaw and, as a result, are characterized by lower strength. Moreover, it is well known that the scale effect is more pronounced if the material is less homogenous [3,21,22]. The homogeneity of concrete is mainly dependent on the distribution of inclusions (aggregate) in the cement matrix, the aggregate size and shape, the difference of the strength and modulus of elasticity of the aggregate and cement matrix, and the bond between these two components. The scale effect is also determined by the geometrical characteristics of the specimens themselves. Due to significant differences in the stiffness of a concrete specimen and compression testing machine platens, in the area of their contact, the uniaxial stress state is disturbed by friction and pressure. As a result, specimens with a larger cross-sectional area exhibit lower strength. At the same time, the shape of the specimen cross-section and its slenderness (*λ* = height (*h*)/cross-section dimension (*d*)) are not insignificant. The circular cross-section provides a more uniform stress distribution compared to the square one because its failure is less affected by the end restraint of the specimen. Moreover, the strength of the cylinders is less influenced by the properties of the coarse aggregate due to the more uniform composition of concrete along the circular edge in comparison to specimens of a square cross-section revealing a higher content of cement paste in the corners. Hence, at the same slenderness and cross-sectional area, cylindrical specimens may exhibit higher strength than cubes [3]. Lowering the specimen slenderness also promotes the strength increase. For ordinary concrete, the typical ratio of strength determined on molded cylinders of *λ* = 2.0 and 1.0 is ca. 0.85–0.95 and is lower for lower strength concrete. The scale effect in the case of normal-weight concrete of different types—plain, ordinary, self-compacting, high strength and ultra-high strength (reactive powder concrete), fiber reinforced—was proven in numerous studies, e.g., [23,24,25,26,27,28,29,30,31,32,33,34]. There are two general conclusions resulting from this research concerning normal-weight concrete: (1) the higher the concrete strength, the lesser the scale effect; (2) the specimen slenderness is the crucial parameter determining the scale effect.

In general, the scale effect of LWAC should be expected to be less pronounced in relation to NWAC because the structure of lightweight aggregate concrete is usually more homogenous in comparison to normal-weight concrete. The main reasons for the better homogeneity of LWAC are the following:

- The more regular shape and size of manufactured aggregates;
- The smaller difference between the values of the strength and modulus of elasticity of porous aggregate and cement matrix;
- The better bond between porous aggregate and cement paste, resulting from better adhesion, absorption of mixing water by the porous aggregate, and in some cases, the pozzolanic reaction.

Confirmation of the less pronounced scale effect of LWAC was found in some research [3,13,35,36,37]. The lower significance of the scale effect in compressive strength tests of lightweight aggregate concrete is reflected also in the strength classification according to European Standard EN 206 [38]. The ratio of the LWAC characteristic strength determined on standard cylinder and cube specimens (*f_ck_*_,*cyl*_/*f_ck_*_,*cube*_), resulting from the strength classes specified in EN 206 [38], ranges from 0.89 to 0.92 and is independent of the concrete strength class. Moreover, the standard states that other values may be used for LWAC if the relationship between the cube and the reference cylinder strength is established and documented. Meanwhile, for NWAC, *f_ck_*_,*cyl*_/*f_ck_*_,*cube*_ ranges from 0.78 up to 0.87 and is higher for higher strength classes. Nevertheless, there are some reports indicating the opposite trends. It was shown in [39,40] that the size effect was stronger in LWAC than in NWAC, and this trend was more pronounced at the specimen slenderness of 2.0 than at that of 1.0. The lateral dimension of specimens also strongly influenced the strength test results of both NWAC and LWAC. On the other hand, it was proven that the size effect was minimally affected by the section shape of the specimen at the same *λ*. Additionally, in the case of LWAC, the aggregate size was not of importance for the scale effect. The probable reason for such a discrepancy in the qualitative assessment of the scale effect of LWAC presented in [39,40] and [3,16,35,36,37] is the type of aggregate. The authors of [39,40] declared that the expanded clay used for the research was characterized by a closed surface with a smooth texture. Such a type of lightweight aggregate could cause a weak bond with cement paste, especially when compared with the crushed granite used for NWAC. Moreover, if the porous aggregate is initially saturated, the adhesion of cement paste may be extremely limited, and the lightweight concrete prepared with such an aggregate should be no longer treated as a material of good homogeneity. 

The basic difference in the scale effect determined for molded and cored specimens consists of lack of the “wall effect” in this last case. Besides, the specimens taken from a structure usually have different, less favorable, conditions of compaction and curing in comparison to molded specimens. Moreover, the process of specimen drilling may itself cause some microcracks in cored specimens. As a result, it is assumed in EN 13791 [41] that for all types of structural concretes, the cored specimens show ca. 15% lower strength than molded ones. Meanwhile, owing to the better structural homogeneity in comparison to normal-weight concrete, LWAC in a structure, even when it is massive, may be less susceptible to cracking resulting from both the drilling process and the temperature increase during the cement hydration. As was shown in [17,18], LWAC, due to better structural homogeneity, revealed lower stress concentration under load and was less susceptible to cracking in comparison to normal-weight concrete. The work in [19], dedicated to the study of the ratio of the initial and stabilized secant moduli of elasticity used as an indicator of concrete susceptibility to microcracking, proved higher resistance of structural lightweight concrete to stress-induced micro-cracking or micro-cracking caused by drilling in comparison to structural normal-weight concrete. On the other hand, there are numerous test reports showing that under high temperature, LWAC performed better than NWAC. For example, the research results presented in [15,16] showed that LWAC subject to temperatures up to 200 °C or even 300 °C, respectively, did not show any microcrack development and strength decrease. Therefore, a higher temperature (up to 90 °C) developed during cement hydration in a structure made of LWAC usually is not able to cause microcracking. Moreover, due to inner curing by water accommodated in porous aggregate, LWAC in a construction usually reveals less sensitivity to external curing conditions in comparison to normal-weight concrete. To sum up, the structure of lightweight aggregate concrete in molded specimens cured in laboratory conditions and in a construction may be less diverse than in the case of normal-weight concrete. Therefore, it may be expected that the difference between strengths determined on LWAC molded and cored specimens would be lower than assumed in EN 13791 [41] for all types of concrete.

Although the European Standard EN 13791 [41] provides principles and guidance for the assessment of the in situ compressive strength of concrete in structures and precast concrete components, it is rather focused on normal-weight concrete, and some specific data resulting from the scale effect are given only for NWAC. Generally, it is assumed that a core diameter ranging from 75 up to 150 mm has no influence on a strength test result. However, the core slenderness affects the achieved value. In the case of normal-weight and heavyweight concrete, the ratio of strength determined on cored cylinders of *λ* = 2.0 and 1.0 may be assumed as 0.82, while for lightweight concrete, there is no relevant information. For LWAC, EN 13791 [41] recommends applying provisions valid in the place of use or to justify some relationships by testing. Such a situation is caused by the lack of sufficient reliable data related to the scale effect of LWAC cored specimens, which is confirmed by the lack of literature reports on this subject. Meanwhile, there are some prerequisites indicating that, as in the case of molded specimens, the scale effect in strength tests of LWAC cored specimens is less significant than in the case of NWAC.

Since there are no specific guidelines for the testing and estimation of lightweight concrete strength in a construction or in precast members, the main purpose of the research was to assess the unrecognized scale effect in compressive strength tests carried out on cored LWAC specimens. An additional aim of the research was to verify whether the assumed strength decrease of 15% for cored specimens in relation to molded ones was valid also for LWAC. For these purposes, four series of lightweight aggregate concrete with a closed structure of different compositions were prepared, and for each series of concrete, both standard molded specimens, as well as 12 types of cored cylinders were tested to determine the compressive strength. The research program carried out enabled quantitative and qualitative assessment of the scale effect of cored LWAC specimens against the background of available data on the effect for normal-weight concrete. It also gave some information on the selection of the types of cored specimens to achieve reliable results of compressive strength of lightweight concrete built into a construction or a precast element. Such information may be of practical importance in the case of the estimation of compressive strength for structural assessment of an existing structure or the assessment of the compressive strength class of LWAC in case of doubt.

## 2. Materials and Methods

**Figure 1:**
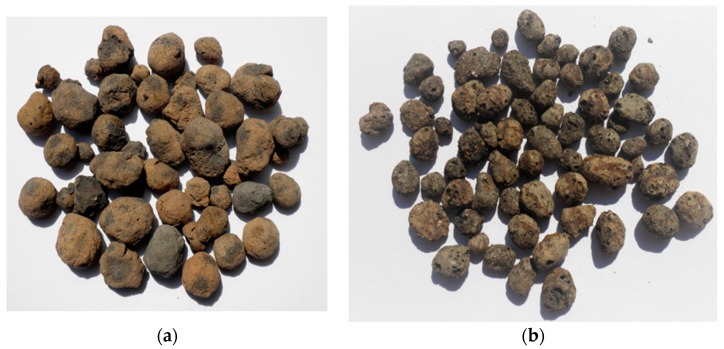
Lightweight aggregates used for concrete to be tested: (**a**) sintered fly ash and (**b**) expanded clay.

The compositions of prepared LWAC differed in the type of lightweight aggregate (LWA) and the strength of cement matrix, as well as their volume share. Two types of coarse lightweight aggregate were selected: expanded clay (EC) and sintered fly ash (SFA) (Figure 1). These types are the most popular porous aggregates used for structural lightweight concrete in the world. However, the expanded clay used in this research was characterized by much lower particle density and a more porous external shell in comparison to the sintered fly ash. Therefore, in practice, such an aggregate is rather used for manufacturing of precast members made of insulating-structural concrete than for typical structural aims. In this research, the application of weak expanded clay aggregate was mainly intended to show the scale effect also in the case of LWAC of lower strength and of less homogeneity in comparison to sintered fly ash aggregate concrete. The basic properties of the applied lightweight aggregates are presented in Table 1. The aggregates before application to concrete were initially moistened to the level corresponding to their absorption after immersion in water for 1 h. Such a moisture content—34.4% and 17.0%, respectively, for expanded clay and sintered fly ash—on the one hand protected the fresh concrete against workability loss and, on the other hand, ensured a good adhesion of the cement paste.

The rest of the constituent materials for concrete mixtures were as follows: Portland cement CEM I 42.5 R, natural sand 0/2 mm as a fine aggregate, tap water, and superplasticizer. Cement mortars, being cement matrices for the prepared lightweight concretes, were characterized by significantly different water /cement ratio (w/c) of 0.55 and 0.37. The share of coarse lightweight aggregate in prepared concretes ranged from 52 up to 55%, respectively, for w/c = 0.37 and 0.55. The concrete compositions are presented in Table 2.

**Figure 2:**
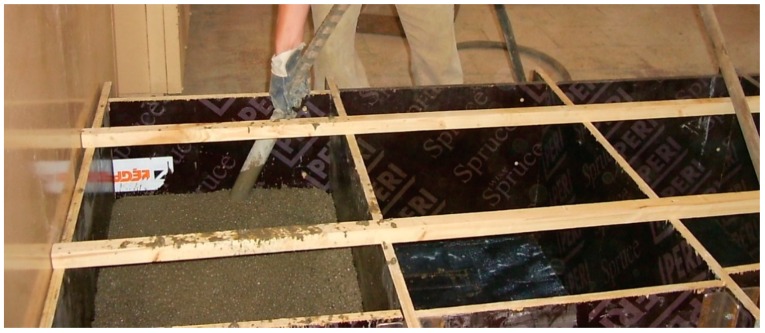
Preparation of concrete blocks for drilling cores.

From each concrete series, 6 standard cubes (d = 150 mm) and 6 cylinders (d = 150 mm and h = 300 mm) were molded as reference specimens. Additionally, for comparative purposes, standard cubes with mortars of compositions corresponding to those used in the concretes were molded. Besides, 4 big concrete blocks with dimensions of 400 × 600 × 1000 mm were cast for drilling cored specimens (Figure 2). Specimens after demolding were stored until the day of test in conditions T = 20 ± 2 °C, RH = 100 ± 5%, meeting the requirements of EN 12390-2 [42]. At the same time, the big blocks were sprinkled with water to ensure similar curing conditions. Nevertheless, on the first days of curing, the temperature of the blocks was much higher than the temperature of standard molded specimens. On the top surface of the blocks, it reached 50 °C and 70 °C, respectively, for concrete Series I and II, due to bigger dimensions of the elements. The inner temperature was certainly even higher.

**Figure 3:**
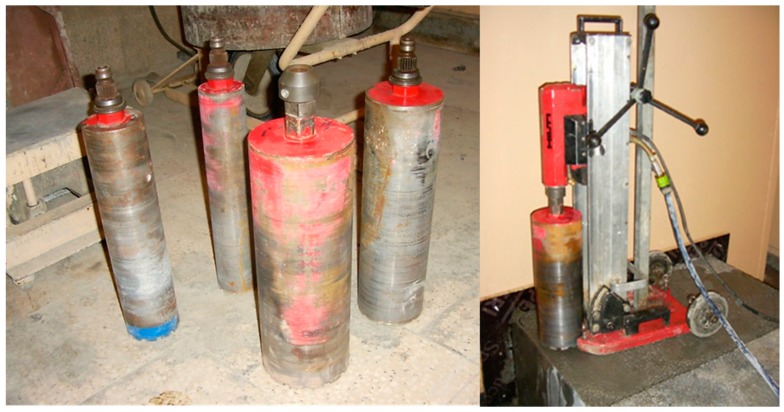
Types of drilling rigs used (d = 80, 100, 125, 150 mm) and cutting out cores from a concrete block.

**Figure 4:**
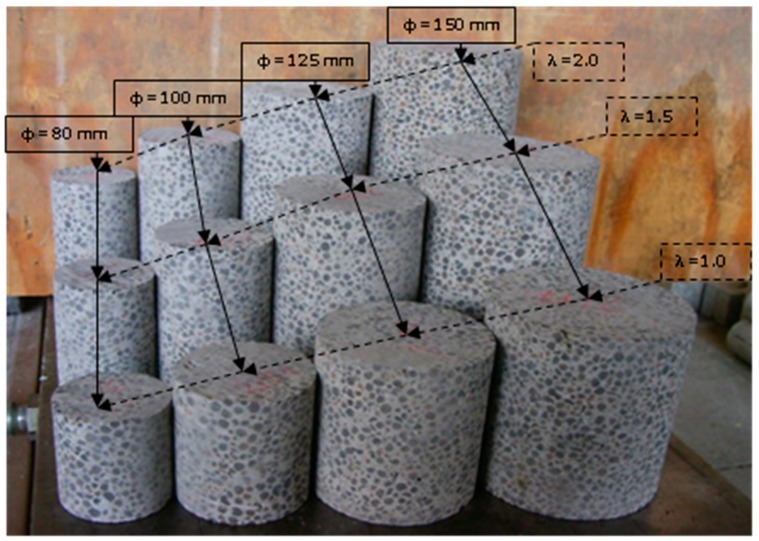
12 types of cored specimens of various diameters *d* and slenderness *λ* to be subject to compressive strength tests.

After 28 days of curing, cores were drilled from the blocks and cut into specimens according to EN 12504-1 [43]. Four drilling rigs of diameters d = 80, 100, 125, and 150 mm were applied (Figure 3). This diameter range is the most commonly used for assessment of in situ compressive strength in structures. The cores were cut into specimens of a slenderness of 1.0 and 2.0, typically used for assessment of in situ compressive strength, and additionally, 1.5. The types and numbers of specimens prepared for tests are presented in Table 3 and Figure 4. Seven cored specimens of a particular type (diameter and slenderness) were cut from each concrete series: 6 as a basic set for the scale effect tests in natural moisture condition (as-received) and 1 for the control test in dry condition. The specimens in the oven-dried condition were mainly applied for the oven-dried density test (the basic one for lightweight concrete), and then, they were additionally used for complementary assessment of the scale effect. In practice, cored specimens drilled from the structure were tested in as-received moisture condition or, if it was required, in a saturated condition. In the case of this research, the specimens’ condition was as-received, but it was very close to the saturated one due to curing. The temperature of specimens’ drying was as low as 50 °C to avoid the risk of concrete microcracking.

The total number of cored specimens to be tested was 336. The density and compressive strength of the moist molded and cored specimens were tested at the age of 28 days according to EN 12390-7 [44] and EN 12390-3 [45], respectively. The dried specimens were tested according to the same procedures, but at the age of 35 days, when they achieved an oven-dried condition.

## 3. Results

The results of the tests carried out on molded specimens are presented in Table 4. The results of density in wet and dry conditions, as well as moisture content tests carried out on cored specimens are presented in Table 5. The values given in Table 5 are the averages determined for a given concrete on a whole set of 72 and 12 cored specimens, respectively, in wet and oven-dried conditions.

**Figure 5:**
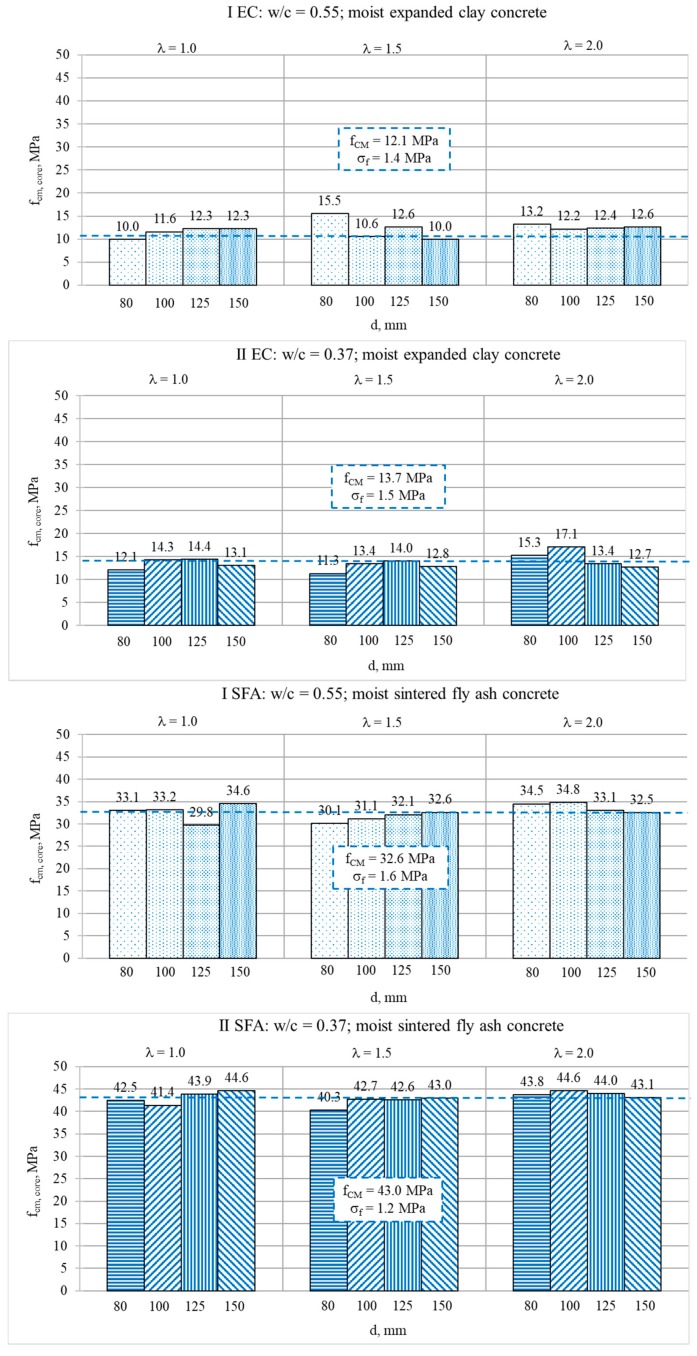
Mean values of compressive strength determined for moist cored specimens of various diameters *d* and slenderness *λ*.

**Figure 6:**
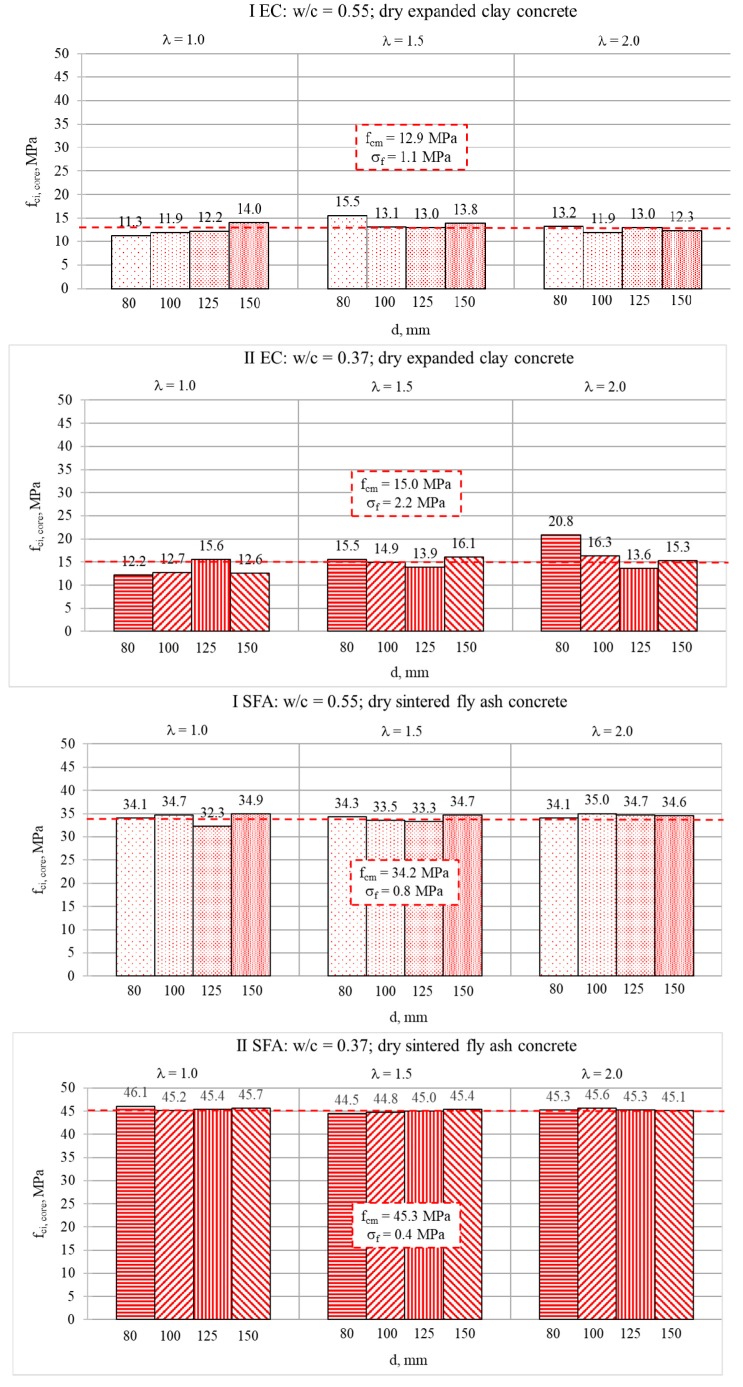
Individual test results of compressive strength determined for dry cored specimens of various diameters *d* and slenderness *λ*.

The results of the compressive strength tests determined for cored specimens are presented in Figure 5 and Figure 6, respectively, in wet and dry condition. It should be noted that mean strength values (*f_cm_*), calculated as the averages of six cores of the same type, are presented in Figure 5. The global mean strength value (*f_CM_*) was calculated as an average of the mean values of all core types. Meanwhile, strength results presented in Figure 6 were determined on single oven-dried specimens. Therefore, these results may be treated only as complementary, and they could not be the basis of the quantitative analyses of the scale effect.

## 4. Discussion

Analysis of the results showed, as assumed, significantly different levels of compressive strength and density of the four concrete series. The concrete strength ranged from 14.5 to 49.5 MPa when determined for molded cube specimens and from 13.8 to 47.6 MPa for molded cylinders. The concrete oven-dried density ranged from 1140 to 1680 kg/m^3^, and in wet condition, the corresponding range was 1290–1880 kg/m^3^. The “wall effect” seemed to have a negligible influence on concrete density; therefore, there were almost no differences between the results determined for molded and cored specimens. Moreover, the similar results of density tests carried out on molded specimens cured in water and cored specimens indicated that the condition of the cores was similar to the saturated due to external curing, but primarily owing to inner curing with water accommodated in porous aggregate. The values of moisture content of concretes were particularly interesting. Despite expanded clay being characterized by water absorption almost twice as high as for sintered fly ash, the moisture content of the tested lightweight concretes seemed to depend mainly on the tightness of the cement matrices. If the aggregates were used initially saturated, their water absorption would certainly affect the water absorption/moisture content of the composites. In the case of the tested concretes, the aggregates were only initially moistened to the moisture content, ensuring a good bond and sealing the aggregate structure with the cement paste. Such an effect was proven in [46].

**Figure 7:**
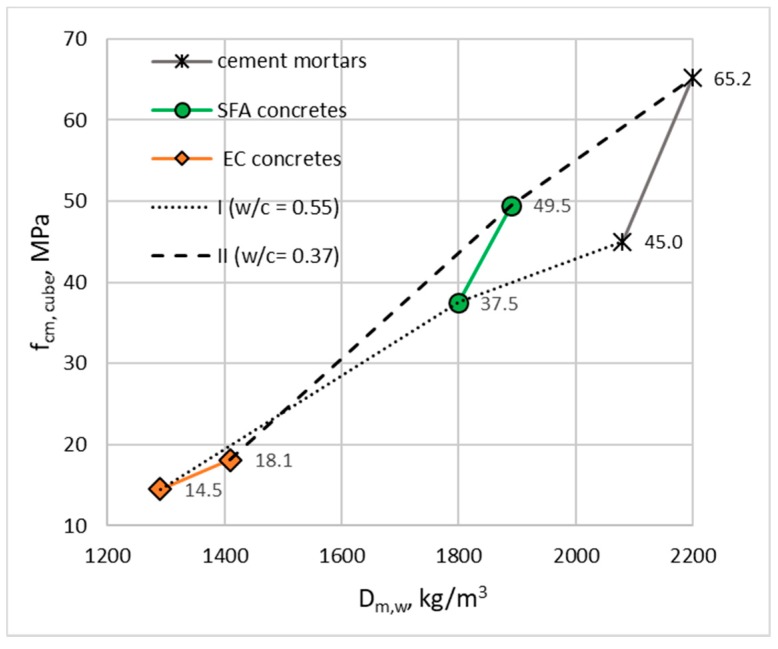
The effect of the application of different cement mortars as matrices for lightweight concretes with sintered fly ash (SFA) and expanded clay (EC) aggregates on their density and strength (wet condition).

Generally, concretes made with stronger, sintered fly ash aggregate (I SFA and II SFA) achieved higher density and compressive strength (almost three times) than those made of expanded clay (I EC and II EC). The strength improvement by the application of a stronger mortar (II of w/c = 0.37) as a cement matrix was also much more effective in the case of SFA concretes than for EC concretes (Figure 7). In the case of the latter concretes, the application of so weak an aggregate limited the possibility of concrete strength increase by increasing cement matrix strength to a large extent. It should be noted that the strength of all lightweight concretes was lower than the strength of the cement mortars used as their matrices, which is typical for LWAC with a closed structure.

The ratio of strength determined on standard cubes and cylinders (*f_cm_*_,*cyl*_/*f_cm_*_,*cube*_) was dependent on concrete homogeneity: The smaller the difference in aggregate and cement matrix strength, the higher the ratio. The mean ratio values were 0.95, 0.93, 0.99, and 0.96, respectively, for concretes I EC, II EC, I SFA, and II SFA. Therefore, these values were clearly higher than those resulting from EN 206 [38] and confirmed the much less pronounced scale and shape effect of the tested lightweight concretes in comparison to normal-weight ones. Especially, it should be noted that concrete II EC with the lowest value of the ratio should not be used at all in practice for material and economic reasons. For the aims of this research, it was prepared of highly strong cement matrix and very weak lightweight aggregate to obtain a lightweight composite of poor homogeneity. There was one more conclusion resulting from the achieved values of the ratio *f_cm_*_,*cyl*_/*f_cm_*_,*cube*_: assessment of lightweight aggregate concrete strength determined for standard cylinders may lead to a higher class than in the case when it is determined for standard cubes.

In the case of the cored specimens, the size effect turned out to be basically imperceptible (Figure 5). This tendency may be observed even in the case of the results of single dry cored specimens (Figure 6). Nevertheless, for obvious reasons, the results achieved on single specimens in dry condition should not be taken into further quantitative analyses of the scale effect. When analyzing the mean strength values presented in Figure 5, it seemed that the type of cored specimens had no impact on the strength result regardless of the type of concrete. As was assumed in EN 13791 [41], the core diameter in the tested range, 80–150 mm, at the given slenderness did not visibly affect the strength results. Moreover, in contrast to NWAC, the slenderness of tested LWAC seemed not to have a noticeable influence on the results either. However, in the case of less homogenous, weaker concretes made with expanded clay, the dispersion of values of mean strength (*f_cm_*) was slightly bigger in comparison to concretes with sintered fly ash. To confirm these observations, a more detailed analysis was carried out. The analysis covered the results’ dispersion for a particular cored specimen type, as well as the ratio of mean strength values determined for the reference cored cylinder (d = 150 mm, h = 300 mm) and a particular cored specimen type.

**Figure 8:**
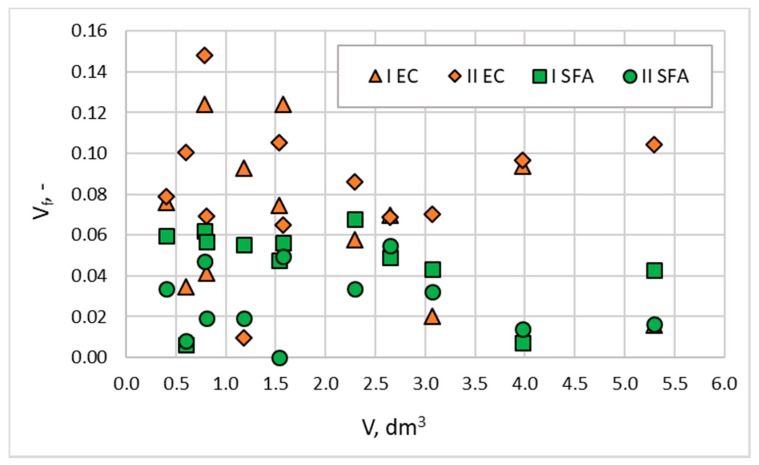
The relationship between cored specimen volume (*V*) and the coefficient of variation of strength determined for particular specimen types (*V_f_*) (wet condition).

The research of strength results dispersion showed that for all tested concretes, the values of standard deviation (*σ_f_*) and coefficient of variation (v_f_ = *σ*_f_ /f_c_) were rather independent of the volume and slenderness of cored specimens. The rule of greater dispersion of the strength test results for specimens of smaller volume was not confirmed here. The coefficients of variation for a particular cored specimen type are presented in Figure 8. The values of *v_f_* ranged from 0.01 to 0.15, and their average values were 0.07, 0.08, 0.05, and 0.03, respectively, for concretes I EC, II EC, I SFA, and II SFA. The values of *σ_f_* for a particular type of cored specimen ranged from 0.3 to 2.2 MPa, and their average values were 1.1 MPa, 0.9 MPa, 1.5 MPa, and 1.2 MPa, respectively, for concretes I EC, II EC, I SFA, and II SFA. These values were almost the same as the standard deviations of the values of mean strength (*f_cm_*) in relation to the global average (*f_CM_*), presented in Figure 5. Such a dispersion convergence suggested that the differences of the results presented in Figure 5 were caused rather by the results’ spread than any scale effect. Very low values of *v_f_* proved the excellent structural homogeneity of the tested lightweight concretes, especially of composites with sintered fly ash aggregate. The results also indicated the possibility of using even the smallest core specimens (within the considered range) to assess strength in a lightweight concrete structure without increasing the number of specimens.

**Figure 9:**
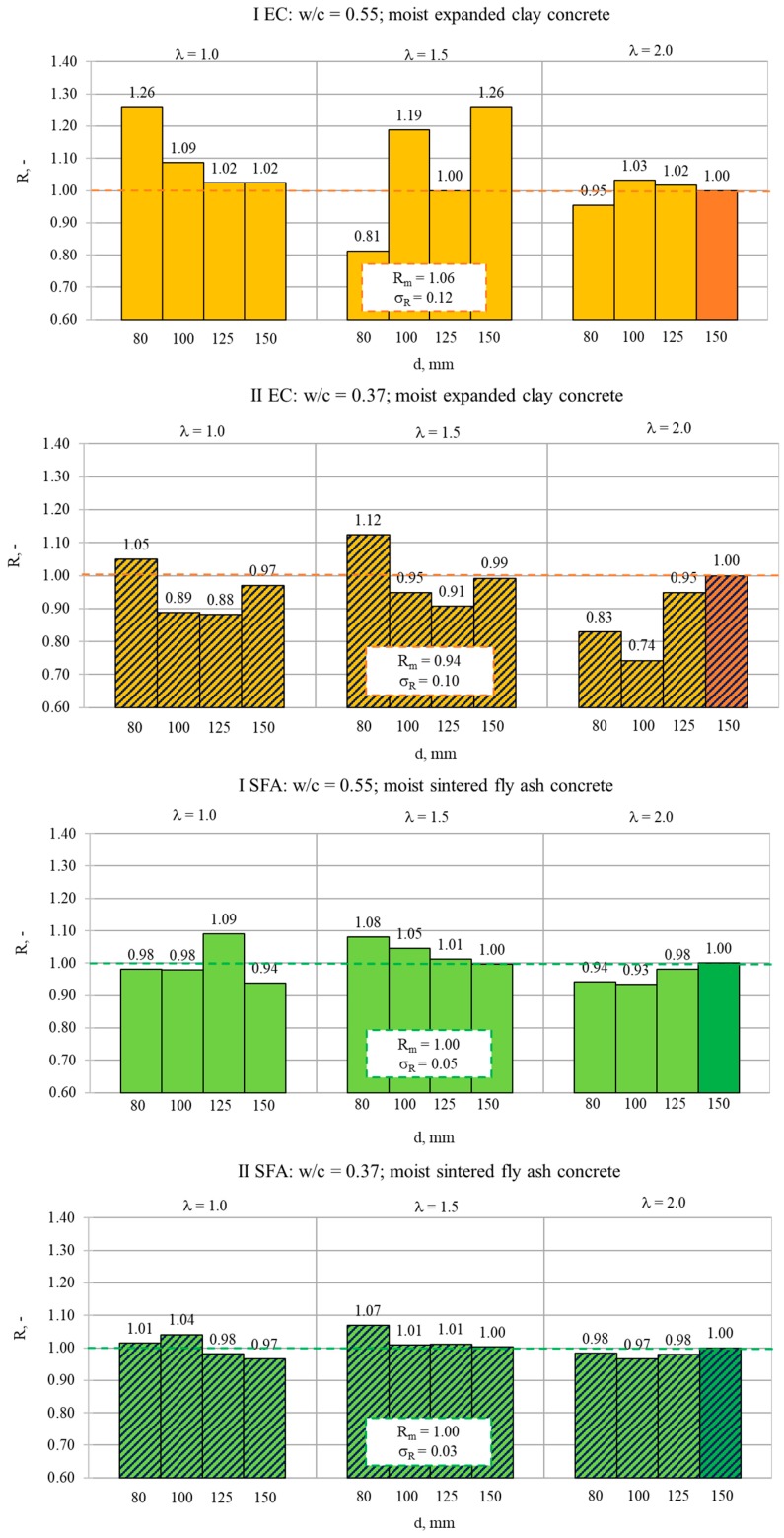
Ratio R = f_cm, 300:150 core_ /f_cm, h:d core_ (wet condition).

The results of the analysis of the ratios of mean strength values determined on the reference cored cylinder (d = 150 mm and h = 300 mm) and on cored specimens of a particular type (R = f_cm, 300:150 core_/f_cm, h:d core_) are presented in Figure 9. They confirmed the much better structural homogeneity of the tested lightweight concretes, especially those made of sintered fly ash aggregate, in relation to normal-weight or heavyweight ones. For all LWACs, the standard core length factor (*f_cm 300:150 core_/f_cm 150:150 core_*) was considerably higher (on average 0.98) than the 0.82 assumed by EN 13791 [41] for normal-weight and heavyweight concretes. For both series of sintered fly ash concretes (I FSA and II FSA), the mean value of the strength ratio *R* equaled exactly 1.00, and no influence of slenderness or core diameter was observed. This means that in the case of such concretes, the type of cored specimens may be assumed as not relevant to in situ strength results. However, in the case of expanded clay concretes, the interpretation of the strength ratio results was not so clear. The mean ratio value was 1.06 and 0.94 for concrete I EC and II EC, respectively, and generally, the dispersion of the ratio values was much bigger in comparison to concretes with SFA. To determine the reliable value of the strength ratio for such weak concretes, some additional verification tests should be carried out.

It should be noted that the cored specimen condition, which is neither specified in EN 12504-1 [43], nor taken into consideration in EN 13791 [41], may affect the assessed strength class of concrete in a measure. Meanwhile, the research also showed that oven-dried cored specimens revealed higher strength by 5% and ca. 8%, respectively, for concretes SFA and EC, than those tested in wet condition. The strength decrease of moist specimens was probably caused more by the considerable moisture content than by earlier age of testing (dry specimens needed an additional seven days besides the standard age of 28 days to dry out).

Despite the demonstrated lack of size and shape effect in the compressive strength tests of lightweight concretes, there were clear differences between the results obtained for molded and cored specimens. The ratio of strength values determined on cored and molded cylinders *f_cm_*_, *core*_*/f_cm_*_, *cyl*_ was 0.91, 0.75, 0.88, and 0.91, respectively, for concretes I EC, II EC, I SFA, and II SFA. The lowest ratio value in the case of concrete II EC may result from its least homogeneity in comparison to the other concretes. As was previously mentioned, such a concrete made of very weak aggregate and strong cement matrix was used in this research only for comparative purposes and should not be applied in practice. The other concretes (I EC, I SFA, and II SFA), which were examples of typical LWAC used for precast member manufacturing or construction execution, revealed a higher ratio *f_cm_*_, *core*_*/f_cm_*_, *cyl*_ (on average 0.90) than that assumed in the standard (0.85). Generally, due to different technologies of LWAC production and various types of structure of lightweight aggregate applied in the world, the ratio value of 0.85 may be retained in the common guidelines for assessment of concrete strength in a structure or a precast element. Nevertheless, in the case of lightweight aggregate concrete of a more homogeneous structure, the overestimation of the strength class of LWAC built in a construction or precast elements should be taken into account. Therefore, the standard recommendation to form provisions valid in the place of LWAC use was fully justified. For tested LWAC, excluding concrete II EC, the “wall effect” and different curing temperature seemed to be the dominant factors determining the difference between strengths specified on cored and molded specimens. Concrete moisture condition (due to inner curing) and susceptibility to microcracking resulting from the drilling process or high temperature probably were of less importance here than in the case of NWAC.

## 5. Conclusions

The research program carried out and the analysis of obtained results revealed no scale effect in compressive strength tests determined on cored specimens of four types of lightweight aggregate concretes with a closed structure. Neither the slenderness, nor the core diameter seemed to affect the strength results. This fact should be explained by the incomparably better structural homogeneity of the tested lightweight concretes in comparison to normal-weight ones. Moreover, the rule of the greater dispersion of the strength test results for specimens of a smaller volume was not confirmed here. This means that, in contrast to NWAC, it was possible to reliably assess the compressive strength of such LWAC types built in a construction or precast components using even the smallest cores (within the considered range) without increasing the number of specimens. Besides, in the case of such concretes, it seemed sufficient to use cores with a slenderness of 1.0 instead of the required 2.0 if the strength test results were to be related to 2:1 molded cylinders. Nevertheless, it should be supposed that in the case of lightweight concrete prepared with initially saturated aggregate or with aggregate particles of tighter and/or smoother external shale, the size effect may be more pronounced. Therefore, the quantitative findings of this research could not be generalized for all types of LWAC.

Despite the demonstrated lack of scale effect in the compressive strength tests of lightweight concretes, there were clear differences between the results obtained on molded and cored specimens. However, for tested LWAC, excluding the concrete II EC, the ratio *f_cm_*_, *core*_*/f_cm_*_, *cyl*_ was slightly higher (on average 0.90) than the 0.85 assumed in the standards. As a result, the application of the standard ratio for compressive strength assessment of an existing structure made of such types of LWAC may lead to overestimation. Therefore, the standard recommendation to form provisions valid in the place of LWAC use was fully justified.

The analyze of the relationship between strength specified on standard molded specimens showed that, due to the much less pronounced scale effect of LWAC in relation to NWAC, assessment of lightweight aggregate concrete strength determined on standard cylinders may lead to a higher strength class than in the case when it is determined on standard cubes.

## Figures and Tables

**Table 1:** Properties of coarse lightweight aggregates.

Aggregate Type	Fraction, mm	Particle Density, kg/m^3^	Water Absorption, %	Crushing Resistance, MPa
Expanded clay	4/8	550	41.2	1.4
Sintered fly ash	4/8	1350	24.3	8.0

**Table 2:** Compositions of mortars and lightweight concretes. LWA, lightweight aggregate; EC, expanded clay; SFA, sintered fly ash.

Series	LWA Type	Nominal w/c	Cement, kg/m^3^	Water, kg/m^3^	Superplast., kg/m^3^	LWA ^1^, kg/m^3^	Sand, kg/m^3^
I mortar	-	0.55	754	415	0.0	-	906
II mortar	-	0.37	912	335	18.4	-	937
I EC	Exp. clay	0.55	338	186	0.0	308	406
II EC	Exp. clay	0.37	446	164	9.0	287	458
I SFA	Sint. fly ash	0.55	338	186	0.0	749	406
II SFA	Sint. fly ash	0.37	446	164	9.0	699	458

^1^ In dry condition.

**Table 3:** The types and numbers of specimens prepared for the tests of each concrete series.

Specimens Type	Diameter/Side *d*, mm	Height *h*, mm	Slenderness *λ = h/d*	Specimens Number
**Molded**				
cube	150	150	1.0	6
cylinder	150	300	2.0	6
**Cored**				
cylinder	150	150	1.0	7
cylinder	150	225	1.5	7
cylinder	150	300	2.0	7
cylinder	125	125	1.0	7
cylinder	125	187.5	1.5	7
cylinder	125	250	2.0	7
cylinder	100	100	1.0	7
cylinder	100	150	1.5	7
cylinder	100	200	2.0	7
cylinder	80	80	1.0	7
cylinder	80	120	1.5	7
cylinder	80	160	2.0	7

**Table 4:** Mean values of compressive strength and density determined on molded specimens.

Series	LWA Type	Nominal *w/c*	Density ^1^ *D_m_*_,*w*_, kg/m^3^	Compressive Strength, *f_cm_*_,*cube*_, MPa	Compressive Strength, *f_cm_*_,*cyl*_, MPa
I mortar	-	0.55	2080	45.0	-
II mortar	-	0.37	2200	65.2	-
I EC	Exp. clay	0.55	1290	14.5	13.8
II EC	Exp. clay	0.37	1410	18.1	16.9
I SFA	Sint. fly ash	0.55	1800	37.5	37.1
II SFA	fly ash	0.37	1890	49.5	47.6

^1^ In wet condition.

**Table 5:** Mean values of concrete density and moisture content determined on cored specimens.

Series	LWA Type	Nominal *w/c*	Density ^1^ *D_m_*_,*w*_, kg/m^3^	Density ^2^ *D_m_*_,*d*_, kg/m^3^	Moisture Content, *mc_m_*, %
I EC	Exp. clay	0.55	1300	1140	14.0
II EC	Exp. clay	0.37	1410	1250	12.8
I SFA	Sint. fly ash	0.55	1790	1570	14.0
II SFA	Sint. fly ash	0.37	1880	1680	11.9

^1^ In wet condition; ^2^ in dry condition.
